# Announcement for the New Year

**Published:** 1881-01

**Authors:** 


WE take pleasure in announcing to our readers that this journal of medicine enters upon a new year with better prospects than ever before. The performance of the promises made in the past is the best guarantee of the excellence of the work which is contemplated for the future. We shall, as heretofore, fill these pages with the original writings of the best men of the profession, at home and abroad, and shall continue to receive correspondence from our esteemed contributors, Drs. H. Osgood, of Boston; C. L. Dana, of New York; and A. Van Harlingen, of Philadelphia, to whom we are under obligations for their excellent letters during the past year. Our foreign correspondence will be conducted by medical men of this city who are abroad—our esteemed correspondent, Dr. Wm. Belfield now acceptably filling this position. The department of original lectures will receive as much attention as heretofore, and we propose to publish with each year, an increasingly large number of these from the lips of the representative teachers of medicine throughout the country. In brief, we desire to announce that neither time nor expense will be spared to keep THE CHICAGO MEDICAL JOURNAL AND EXAMINER in the front rank of the medical periodicals of the United States.

